# Integrated analysis and single-cell sequencing of mitochondrial metabolism related gene molecular subtype and diagnostic model in ulcerative colitis

**DOI:** 10.1371/journal.pone.0320010

**Published:** 2025-03-28

**Authors:** Li Li, Xiaoyao Chen, Tao Li, Bing Sun, Bo Zhang, Weifeng Zhang, Junbo Wu, Meng Cui, Guoliang Wu

**Affiliations:** 1 Department of Endocrinology, The Affiliated Hospital of Shandong University of Traditional Chinese Medicine, Jinan, China; 2 Department of Anorectal Section, The First Affiliated Hospital of Shandong First Medical University, Jinan, China; 3 Department of Anorectal Section, The Affiliated Hospital of Xuzhou Medical University, Xuzhou, China; 4 Department of Colorectal Surgery, Hengyang Central Hospital, Hengyang, China; The First Affiliated Hospital of Wenzhou Medical University, CHINA

## Abstract

Ulcerative colitis (UC) is a chronic inflammatory bowel disease that seriously affects the life expectancy of patients. Although increasingly sophisticated combinations of drugs can alleviate symptoms, 10–20% of patients still do not respond well. Therefore, it is necessary to further explore the pathogenesis and potential biomarkers of UC. Many clues have suggested the important value of mitochondrial metabolism in UC, but its role and related targets need to be further explored. By public database data, we identified differentially expressed mitochondrial metabolism related genes (MMRG) in UC. Subsequently, we identified biomarkers associated with MMRG based on a machine learning approach. After classifying the MMRG-associated molecular subtypes of UC, we comprehensively analyzed the MMRG biomarkers and the relationship between the MMRG molecular subtypes and immune infiltration characteristics. Single-cell sequencing analysis showed significant expression pattern of MMRG signatures in different cell subtypes. qRT-PCR and western blot further confirmed the abnormal expressions of selected genes *in vitro.* Our findings provided a new perspective on the role of MMRG in UC.

## Introduction

Ulcerative colitis (UC) is a chronic inflammatory bowel disease (IBD). In 2023, the global prevalence of UC is estimated at 5 million cases, and the global incidence is increasing [1]. In addition to cause chronic diarrhea and rectal bleeding, which reduces patients’ quality of life, UC also increases the risk of colorectal cancer and colectomy, leading to a lower life expectancy [2]. Despite a wider range of treatment options are applied, including 5-aminosalicylic acid drugs, corticosteroids and small molecule drugs, 10–20% of patients still respond poorly [3]. Therefore, it is necessary to further explore the pathogenesis and potential biomarkers of UC.

As an important biological energy hub, mitochondrial metabolism mainly includes the TCA cycle, fatty acid oxidation (FAO), electron transport chain (ETC), and oxidative phosphorylation (OXPHOS) [4]. These biological processes not only participate in the catabolism and energy production, but also provide a variety of biomolecular precursors and adapt to metabolic changes under different disease conditions by modifying nuclear transcription [5]. For example, during the ETC process, escaping electrons interact with oxygen to produce reactive oxygen species (ROS). As a secondary messenger, ROS plays a crucial role in regulating metabolic responses and homeostasis inside and outside the mitochondria [6]. In addition, by affecting the function of Treg and other immune components, the implications of mitochondrial metabolism for immunotherapy and disease treatment also provide valuable insights into novel therapeutic strategies [7,8].

There is a range of evidence suggesting the role of mitochondrial metabolism in UC patients. A genetic study of UC patients showed overexpression of genes involved in inflammation and decreased expression of genes involved in mitochondrial respiration in UC patients [9]. The activity of mitochondrial complexes II, III, and IV was reduced by 50% to 60% in the colon mucosa of UC patients [10]. After treatment in an animal model of IBD, levels of many proteins involved in mitochondrial function were down-regulated in colon tissue compared to untreated animals, also suggesting the potential value of mitochondrial metabolism in UC therapy [11]. The role of mitochondrial metabolic processes in the regulation of immune status in mucosa has also been reported [12]. These clues suggest the important value of mitochondrial metabolism in UC, which needs to be further explored.

In this study, we applied public database data to identify UC disease-related characteristic gene modules. The differentially expressed MMRG in UC were identified. Subsequently, we identified MMRG-related biomarkers based on machine learning methods. After classifying the MMRG-associated molecular subtypes of UC, we conducted a comprehensive analysis of MMRG biomarkers and the association of MMRG molecular subtypes with immune infiltration characteristics. Through single-cell sequencing technology, significant expression of MMRG signatures was observed in different cell subtypes. Our results provide a new perspective on the role of MMRG in UC.

## Methods

### Preprocessing of gene transcriptome features

Using the GEO database, we searched for datasets related to UC with the keyword “ulcerative colitis” and four datasets containing both normal control (NC) and UC samples were included: GSE48634 (NC: 69, UC: 68; Platform: GPL10558), GSE92415 (NC: 21, UC: 162; Platform: GPL13158), GSE107499 (UC: 119; Platform: GPL15207), and GSE179285 (NC: 31, UC: 55; Platform: GPL6480). A total of 121 NC samples and 404 UC samples were extracted for downstream analysis. Gene annotations were performed using platform annotation files in the Perl programming environment. The “sva” package was applied to eliminate batch effects and normalize transcriptome data across datasets, ensuring consistency for subsequent analyses.

### Identification and differential expression analysis of MMRG

Using “MMRG” as a keyword, we retrieved MMRG gene signatures from the GeneCards database (https://www.genecards.org/) (S1 Table). Differential expression analysis between NC and UC groups was conducted using the “limma” package, with screening thresholds set at |Fold Change (FC)| >  1 and *p*.adjust <  0.05 [13–16]. Pathway enrichment analysis was performed using the “GSEA” package, and the normalized enrichment score (NES) was calculated based on differential gene expression. Disease trait-associated differential MMRG signatures were identified using the “venn” package. Potential molecular mechanisms of differentially expressed MMRG were explored using the “clusterProfiler” package, with functional enrichment analyzed through Gene Ontology (GO) and Kyoto Encyclopedia of Genes and Genomes (KEGG) pathway entries.

### Weighted gene co-expression network analysis (WGCNA) model development

Using the transcriptome features of NC and UC samples, we constructed a WGCNA model with the “WGCNA” package to identify gene modules most associated with clinical traits. Initially, a whole-sample clustering dendrogram was built to detect and exclude outliers. The optimal soft-thresholding power was determined through network topology analysis. With the minimum module size set to 50, hierarchical clustering was performed to identify gene modules, followed by dynamic tree cutting and integration. Modules were further clustered with a height cutoff of 0.25 to refine the module selection. Pearson correlation analysis was used to evaluate relationships between module eigengenes and module members, as well as the association between modules and clinical traits. The module exhibiting the highest correlation with clinical traits was identified as the key gene module for subsequent analyses.

### Selection of optimal machine learning algorithms and identification of MMRG biomarkers

Using the expression profiles of characteristic MMRG gene signatures, multiple machine learning algorithms were evaluated to determine the optimal method for identifying MMRG biomarkers. In the R environment, residual boxplots and distribution curves were generated for each algorithm. Based on ROC curve analysis, random forest (RF) and Support Vector Machine Recursive Feature Elimination (SVM-RFE) algorithms with the highest area under the curve (AUC) were selected. The “randomForest” package was used to calculate and rank the importance of MMRG signatures, with variables showing importance > 1.5 identified as key features. Using the “e1071,” “kernlab,” and “caret” packages, an SVM-RFE model was constructed, and cross-validation was performed to calculate the minimum smallest root mean square error (RMSE). Characteristic MMRG variables were identified based on the minimum RMSE values.

### Evaluation of diagnostic efficacy of MMRG biomarkers and nomogram diagnostic model development

The intersection genes from the RF and SVM-RFE machine learning algorithms were identified as MMRG biomarkers. Using the “rms” package, we calculated the coefficients for each biomarker and developed a nomogram diagnostic model with the formula: DUOX2 *  0.516 +  MMP7 *  0.039 +  LCN2 *  0.0567 +  UBE2L6 *  0.129. The “pROC” package was utilized to calculate the AUC of MMRG biomarkers and nomogram scores, and ROC curves were plotted to assess the diagnostic efficacy of each variable. Calibration and decision curves analysis (DCA) were generated using the “rmda” package to evaluate the accuracy and predictive performance of the nomogram diagnostic model.

### Recognition of MMRG molecular subtypes and evaluation of immune infiltration characteristics

Using the expression profiles of MMRG biomarkers, an unsupervised consensus clustering analysis of UC samples was performed with the “ConsensusClusterPlus” package to identify MMRG molecular subtypes. Molecular subtypes were determined by setting the maximum cluster number (K) from 2 to 9 and selecting the optimal k value based on parameters such as consensus CDF and delta area. Principal component analysis (PCA) plots of MMRG molecular subtypes were generated using the “ggplot2” package to assess their independence. Differential KEGG pathway expression between subtypes was analyzed using the “GSVA” package. Additionally, single-sample Gene Set Enrichment Analysis (ssGSEA) analysis was performed to quantify the proportions of 23 immune-infiltrating cell types in each sample, based on immune cell gene signatures, using the “GSVA” package.

### Preprocessing and analysis of single-Cell RNA sequencing data

Single-cell RNA sequencing data for UC were obtained from the GSE231993 dataset. Data importation and preprocessing were conducted in the R environment using the “Seurat” package. Gene expression matrices were normalized, and low-quality cells were filtered out by excluding cells with fewer than 200 detected genes or more than 5% mitochondrial gene content. Highly variable genes were identified and used for PCA, selecting the top 20 principal components for further analysis. Cell clustering was performed with the Louvain algorithm using a multi-scale approach, and 2D UMAP plots were generated to visualize the distribution of cell populations. Subpopulation characteristics were further analyzed through differential gene expression analysis and annotated using the “SingleR” package. Spatial expression patterns of genes within subpopulations were visualized via UMAP and t-SNE diagrams.

### Construction of cell line model and real-time quantitative PCR analysis

The NCM460 cell line was cultured in RPMI-1640 medium (SH30809; HyClone, USA) supplemented with 10% fetal bovine serum (FBS, Hangzhou, China). To establish an in vitro UC model, NCM460 cells were treated with 1 μg/mL lipopolysaccharide (LPS, Sigma) for 24 hours, after which cells were collected for analysis. Total RNA was extracted using Trizol reagent (Cat#9109; TaKaRa, Japan), and cDNA was synthesized with the PrimeScript RT Master Mix kit (Cat#RR047A; TaKaRa, Japan). Real-time quantitative PCR was performed using SYBR Green PCR Mix (Cat#RR420A; TaKaRa, Japan). Data were analyzed using the 2^-ΔΔCT method, with all experiments independently repeated three times (S2 Table).

### Western blot analysis

The NCM460 cell and LPS-induced NCM460 cell were washed with cold PBS and lysed in RIPA buffer (Beyotime, China) containing protease inhibitors (Beyotime, China). The protein concentration was determined using the BCA Protein Assay Kit (Beyotime, China) according to the manufacturer’s instructions. Equal amounts of protein (30 µg) from each sample were separated by 10% SDS-PAGE and transferred onto a polyvinylidene fluoride (PVDF) membrane (Millipore, USA). The membranes were blocked with 5% non-fat milk in TBST (Tris-buffered saline with 0.1% Tween-20) for 1 hour at room temperature. The following primary antibodies were used: DUOX2 (1:1000, Abcam, UK), UBE2L6 (1:1000, Abcam, UK), LCN2 (1:1000, Abcam, UK), MMP7 (1:1000, Abcam, UK), and GAPDH (1:5000, Abcam, UK) as the internal control. Membranes were incubated overnight at 4°C with the primary antibodies, followed by washing and incubation with horseradish peroxidase (HRP)-conjugated secondary antibodies (1:5000, Beyotime, China) for 1 hour at room temperature. Protein bands were visualized using the ECL chemiluminescence detection kit (Beyotime, China) and quantified using ImageJ software.

### Statistical analysis

Data preprocessing and visualization were performed using R and Perl programming languages. The correlation between MMRG biomarkers and immune-infiltrating cells was assessed using the Pearson correlation coefficient. The statistical differences between two groups were evaluated using the Wilcoxon rank-sum test and Student’s t-test, while differences between multiple groups were tested using the ANOVA method. A multiple correction was applied, and a *p*-value of < 0.05 was considered statistically significant, with significance levels defined as * *p* <  0.05, **p <  0.01, and ***p <  0.001.

## Results

### Gene modules for identifying disease-related features in UC

**Fig 1:**
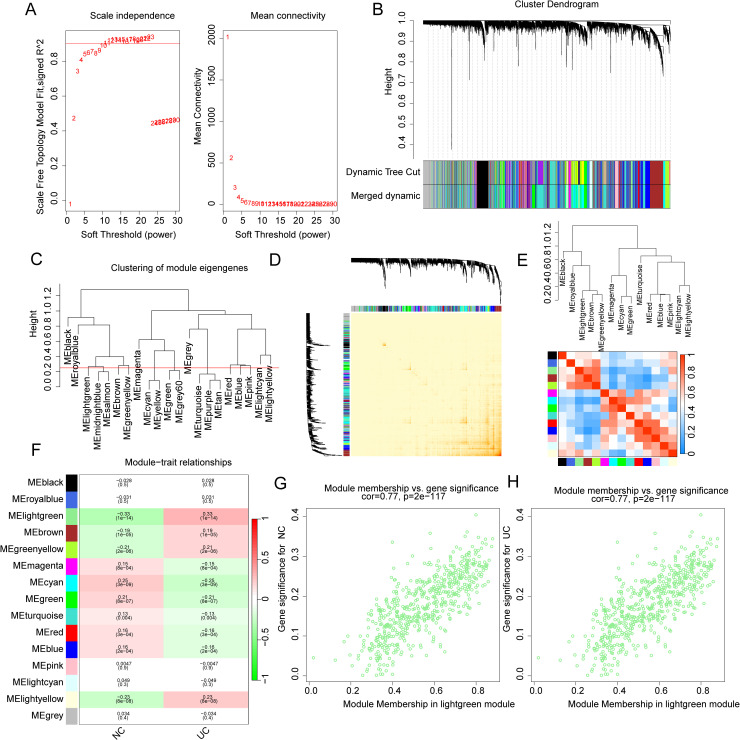
WGCNA model construction. **(A)** Soft threshold identification under the condition that the scale-free topology model fitting parameter R^2 was greater than 0.85. **(B)** Dynamic tree cutting and integrate gene module members. **(C)** Clustering tree analysis of gene module members. **(D)** Association analysis of genes in module members. **(E)** Analysis of potential associations between module members. **(F)** Correlation analysis of gene module members and clinical traits. (**G, H)** Scatter plot analysis of correlation between module membership and gene significance.

In this study, we integrated four independent datasets (GSE48634, GSE92415, GSE107499 and GSE179285) containing both NC and UC samples to explore potential genetic signatures associated with UC. Using 121 NC samples and 404 UC samples derived from these datasets, we constructed a WGCNA model to identify gene modules most associated with the disease. Based on the transcriptomic features of each sample, we first constructed a sample clustering tree to filter out and exclude abnormal samples. To construct the free network, we selected a soft threshold (β=10) ensuring that the scale-free topology model fitting parameter R^2^ exceeded 0.85 (Fig 1A). With the minimum gene module size set to 50, we generated various gene modules and visualized their height using a cluster tree plot. We then used dynamic tree cutting to segment and integrate these gene modules for further analysis (Fig 1B). After setting the height of the cluster tree at 0.25, we performed cluster analysis on the gene modules obtained from dynamic tree cutting and identified 14 significant gene modules for the final analysis (Fig 1C). The heatmap visualization of the network indicated no significant correlation between genes within each module member (Fig 1D). Correlation analysis of module members revealed that gene modules were independent, showing no significant correlations between them (Fig 1E). Pearson correlation analysis was performed to explore the association between gene modules and disease traits. The results indicated that the light-green module was significantly negatively correlated with NC but positively correlated with UC, suggesting it may be the most relevant gene module for UC (Fig 1F). Scatter plot analysis of module members and gene significance in the light-green module revealed a high correlation (r =  0.77, *p* =  2e-117) (Fig 1G, H). These findings suggest that the light-green module could represent a key molecular feature in UC and warrants further investigation for potential biomarkers.

### Identification and potential mechanism analysis of differentially expressed MMRG signatures in UC

**Fig 2:**
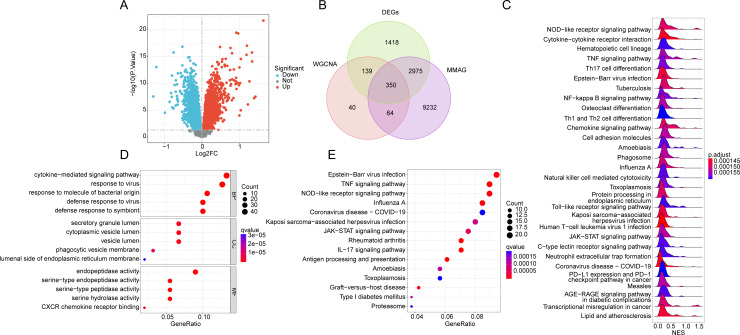
Identification of key UC-related MMRG gene signatures and analysis of potential molecular mechanisms. **(A)** Differential gene signature identification between NC group and UC group, the threshold was set as |FC|>1 and *p*. adjust < 0.05. **(B)** Identification of critical differential expression MMRG signatures. **(C)** GSEA enrichment analysis based on gene expression between UC and NC groups. (**D**, **E)** GO and KEGG enrichment analysis of key MMRG gene signatures with differential expression.

To explore the differential expression of MMRG signatures between NC and UC groups, we first performed differential gene expression analysis. The screening threshold was set at |FC| >  1 and *p.*adjust <  0.05. Differential expression analysis revealed a set of genes that were significantly differentially expressed between the two groups (Fig 2A). Using Venn diagrams, we identified 350 MMRG signatures that were differentially expressed between the UC and NC groups (Fig 2B). Gene Set Enrichment Analysis was conducted to identify the potential molecular mechanisms associated with these differential expressions. The results suggested that immune and inflammation-mediated signaling pathways were significantly upregulated in the UC group. These pathways included the NOD-like receptor signaling pathway, cytokine-cytokine receptor interaction, hematopoietic cell lineage, TNF signaling pathway, Th17 cell differentiation, and Epstein-Barr virus infection (Fig 2C). To further elucidate the potential regulatory mechanisms, we performed GO and KEGG enrichment analysis on the differentially expressed MMRG signatures. The GO analysis revealed that MMRG genes were primarily involved in cytokine-mediated signaling pathways, response to viruses, secretory granule lumen, and endopeptidase activity. KEGG analysis highlighted that MMRG genes were most associated with Epstein-Barr virus infection, the TNF signaling pathway, the NOD-like receptor signaling pathway, and influenza A (Fig 2D, E). From these analyses, we concluded that MMRG signatures play a potential role in the pathogenesis of UC, with immune and inflammation-related signaling pathways being crucial mechanisms through which MMRG may mediate the disease development.

### Identification of MMRG gene characteristics based on machine learning algorithms

**Fig 3:**
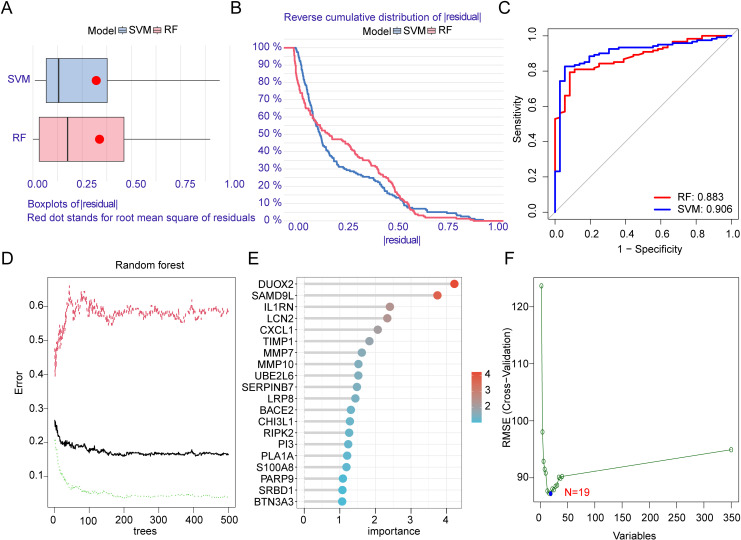
Screening MMRG gene variables associated with UC based on the optimal machine learning model. **(A)** Residual boxplots of different machine learning. **(B)** Cumulative residual distribution curves for different machine learning. **(C)** ROC curve analysis of RF and SVM-RFE algorithms. (**D, E)** Identification of MMRG feature variables based on RF algorithm. **(F)** Identification of MMRG feature variables based on SVM-RFE algorithm.

To identify the characteristic MMRG gene signatures associated with UC, we applied various machine learning algorithms to analyze the 350 differentially expressed MMRG. The goal was to determine the optimal machine learning algorithm for this task. First, we assessed the fitting ability of multiple algorithms by calculating residual plots and cumulative residual distribution curves for each machine learning method (Fig 3A, B). We then performed ROC curve analysis to evaluate the diagnostic accuracy of the algorithms. The AUC values for the RF and SVM-RFE algorithms were 0.883 and 0.906, respectively, indicating high accuracy in identifying UC-related features (Fig 3C). Based on the RF algorithm, nine MMRG gene signatures were identified as potential characteristic variables for UC (Fig 3D, E). Additionally, the SVM-RFE algorithm revealed that the model had the RMSE when 19 variables were included, suggesting that these 19 MMRG genes might be key biomarkers for UC (Fig 3F).

### Identification of MMRG-related biomarkers and construction of nomogram model

**Fig 4:**
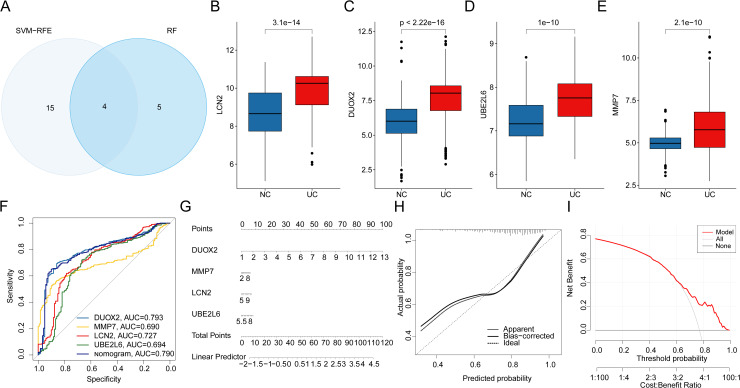
Identification of MMRG-related biomarkers in UC and construction of a nomogram prediction model. **(A)** Identification of MMRG biomarkers based on machine learning algorithms. (**B-E)** Expression analysis of DUOX2, UBE2L6, LCN2 and MMP7 in NC and UC groups. **(F)** ROC curve analysis of MMRG biomarkers and nomogram prediction model. **(G)** Nomogram diagnosis and prediction model based on MMRG biomarker expression profile. (**H**, **I)** Calibration curve and DCA curve analysis of nomogram model.

Using the SVM-RFE and RF machine learning algorithms, we identified four key MMRG intersection genes, which were selected as biomarkers for UC (Fig 4A). Expression analysis revealed that DUOX2, UBE2L6, LCN2, and MMP7 were significantly overexpressed in the UC group compared to the NC group, suggesting that these four MMRG markers are closely associated with UC (Fig 4B-E). Based on the expression profiles of these four MMRG biomarkers, we constructed a nomogram prediction model to assess the diagnostic efficacy of both the individual biomarkers and the composite model. The ROC analysis indicated that the AUC for DUOX2, UBE2L6, LCN2, MMP7, and the nomogram were 0.793, 0.690, 0.727, 0.694, and 0.790, respectively, reflecting satisfactory diagnostic performance (Fig 4F, G). Further evaluation of the nomogram model using calibration and DCA curves demonstrated that the nomogram, based on these MMRG biomarkers, showed good accuracy and predictive ability for diagnosing UC (Fig 4H, I). This model could serve as a valuable tool for clinical diagnosis, offering a more precise approach to assessing UC risk and guiding treatment decisions.

### Identification of molecular subtypes characterization associated with MMRG in UC

**Fig 5:**
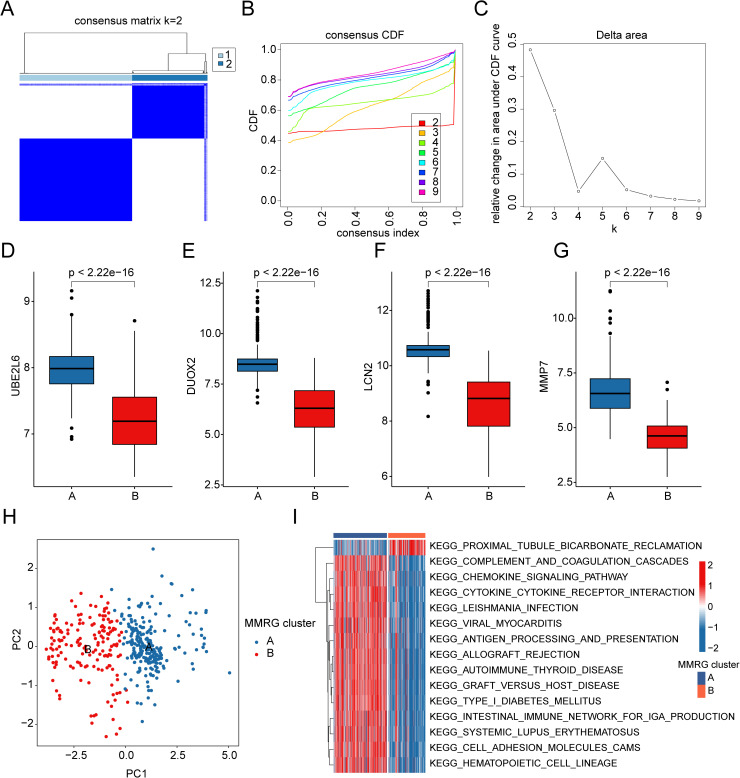
Characterization of MMRG molecular subtypes in UC samples. (**A**-**C)** Characterization of molecular subtypes based on MMRG gene signatures. (**D**-**G)** Differential expression analysis of DUOX2, UBE2L6, LCN2 and MMP7 in MMRG subtypes. **(H)** PCA pattern classification of MMRG molecular subgroups. **(I)** KEGG signal prediction analysis based on MMRG molecular subtypes.

To explore the molecular subtypes associated with MMRG in UC, we performed consensus cluster analysis using the expression profiles of MMRG biomarkers. The analysis revealed two distinct molecular subtypes of MMRG in UC samples. MMRG subtype A consisted of 242 UC samples, while subtype B included 162 UC samples (Fig 5A-C). Further analysis of gene expression profiles revealed that in MMRG subtype B, the expression levels of DUOX2, UBE2L6, LCN2, and MMP7 were significantly down-regulated in comparison to MMRG subtype A (Fig 5D-G). PCA of the molecular subtypes showed clear separation and distinct independence between the two groups, indicating that the subtypes were biologically different (Fig 5H). To understand the functional implications of these subtypes, we conducted GSVA to identify differentially regulated KEGG signaling pathways. The results indicated that the proximal tubule bicarbonate reclamation pathway was significantly up-regulated in MMRG subtype B, suggesting a potential metabolic or renal involvement. Conversely, immune-related signaling pathways, such as the chemokine signaling pathway, cytokine-cytokine receptor interaction, intestinal immune network for IgA production, and cell adhesion molecules, were significantly down-regulated in subtype B. This suggests that immune signaling might be suppressed in the subgroups with higher MMRG expression levels (Fig 5I). Based on these findings, we propose that UC samples can be classified into different molecular subgroups based on the expression of MMRG biomarkers. These subgroups may reflect distinct underlying molecular mechanisms, particularly in relation to immune function and signaling, and could help guide personalized treatment strategies in UC.

### Analysis of potential associations between MMRG biomarkers and immunoinfiltration characteristics

**Fig 6:**
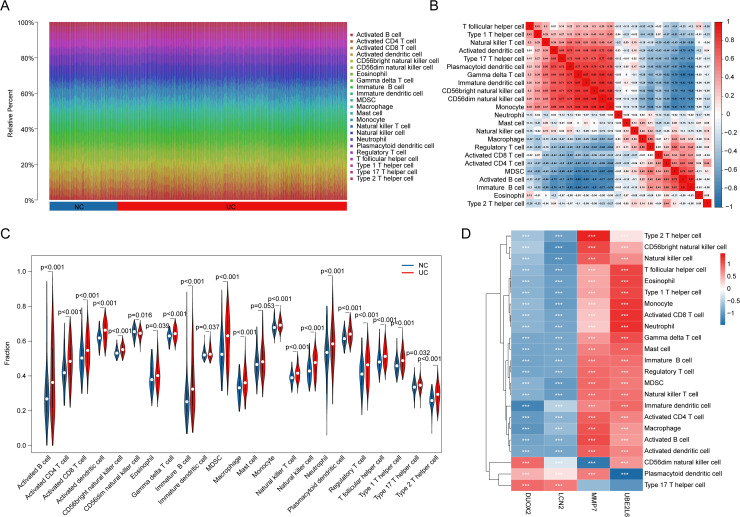
Analysis of the association between MMRG biomarkers and immunoinfiltration characteristics. **(A)** The characteristics of immune cell infiltration in NC and UC groups were evaluated based on ssGSEA analysis algorithm. **(B)** Association analysis of immune-infiltrating cells. **(C)** Quantitative analysis of 23 kinds of immune-infiltrating cells. **(D)** Association analysis of MMRG biomarkers and immune-infiltrating cell characteristics.

In subsequent studies, we further evaluated the immunoinfiltration profile between the NC and UC groups and explored the potential link between the MMRG biomarker and the immunoinfiltration profile. Based on ssGSEA analysis, we evaluated infiltration characteristics between 23 immune cells based on gene expression characteristics in the NC and UC groups (Fig 6A). Correlation analysis of immune cells demonstrated a high degree of correlation among 23 immunoinfiltration features (Fig 6B). Quantitative results of immune infiltration characteristics indicated that the proportion of immune cells infiltrated in UC group was significantly higher than that in NC group, such as activated B cell. activated CD4^+^ T cell, activated CD8^+^ T cell, and activated dendritic cell indicate the highly immune-activated state of UC samples (Fig 6C). In addition, we further evaluated the potential association between the MMRG biomarker and 23 immunoinfiltration features. The results showed that DUOX2 was associated with CD56dim natural killer cell. Plasmacytoid dendritic cell was positively correlated with Type 17 T helper cell and negatively correlated with other immune cells. LCN2 was positively correlated with plasmacytoid dendritic cell and Type 17 T helper cell, but negatively correlated with other immune cells. UBE2L6 and MMP7 were significantly positively correlated with most immune cells (Fig 6D).

### Immunoinfiltration characteristics and molecular mechanism analysis of MMRG subtypes

**Fig 7:**
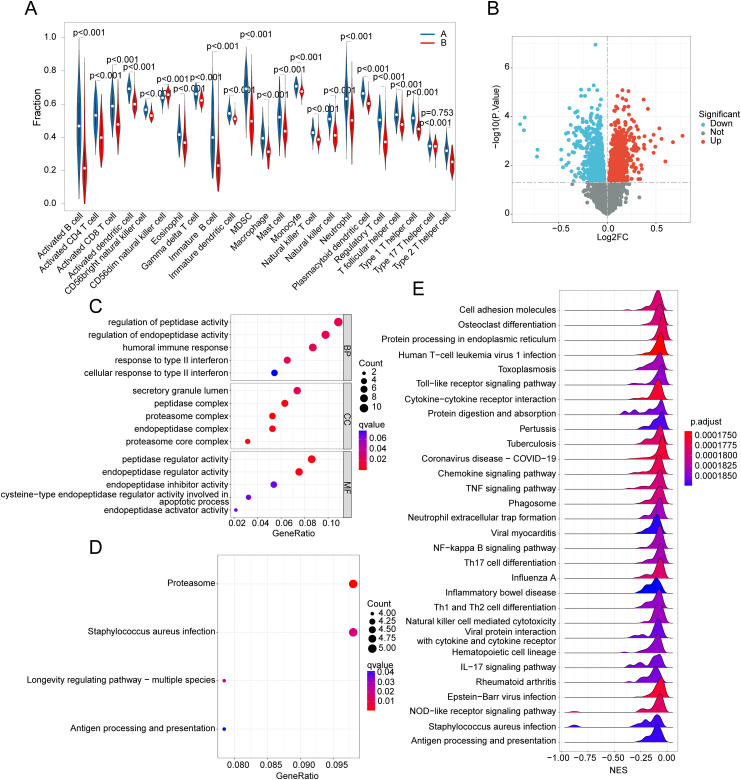
Evaluation of immunoinfiltration characteristics of MMRG subtypes and analysis of potential molecular mechanisms. **(A)** Assessment of immunoinfiltration characteristics of MMRG molecular subtypes. **(B)** Differential expression analysis of MMRGH subtype genes. (**C**, **D)** GO and KEGG prediction analysis of differentially expressed genes of MMRG molecular subtypes. **(E)** GSEA analysis based on sequencing of MMEG subsets.

To further evaluate immune infiltration, we conducted ssGSEA based on the gene transcriptome signatures of UC samples. The analysis revealed that the proportion of immune cell infiltration in MMRG subtype A was significantly higher than in MMRG subtype B. This was particularly evident for activated immune cells, such as activated B cells, activated CD4^+^ T cells, activated CD8^+^ T cells, and activated dendritic cells, indicating a higher level of immune infiltration in MMRG subtype A (Fig 7A). Next, differential gene expression analysis between the MMRG molecular subtypes was conducted, with the threshold set at |FC| >  1 and *p*.adjust <  0.05. The results from GO analysis suggested that the differentially expressed genes between the MMRG subtypes were primarily involved in regulating peptidase activity, endopeptidase activity, humoral immune responses, and secretory granule lumen function (Fig 7B). KEGG pathway analysis pointed to the proteasome pathway and Staphylococcus aureus infection as key regulatory mechanisms associated with these subtypes (Fig 7C, D). Further investigation using GSEA of the molecular mechanisms of MMRG subtypes revealed that immune and inflammation-related signaling pathways were significantly up-regulated in MMRG subtype A. These pathways included cell adhesion molecules, human T-cell leukemia virus 1 infection, toll-like receptor signaling, cytokine-cytokine receptor interaction, and TNF signaling pathways, which aligned with the observed immune infiltration patterns (Fig 7E). Based on these findings, we conclude that there are significant differences in immune infiltration characteristics between the MMRG molecular subtypes of UC. This underscores the potential for tailoring therapeutic strategies based on the molecular and immune profiles of UC patients.

### Single-cell sequencing analysis of MMRG diagnostic markers

**Fig 8:**
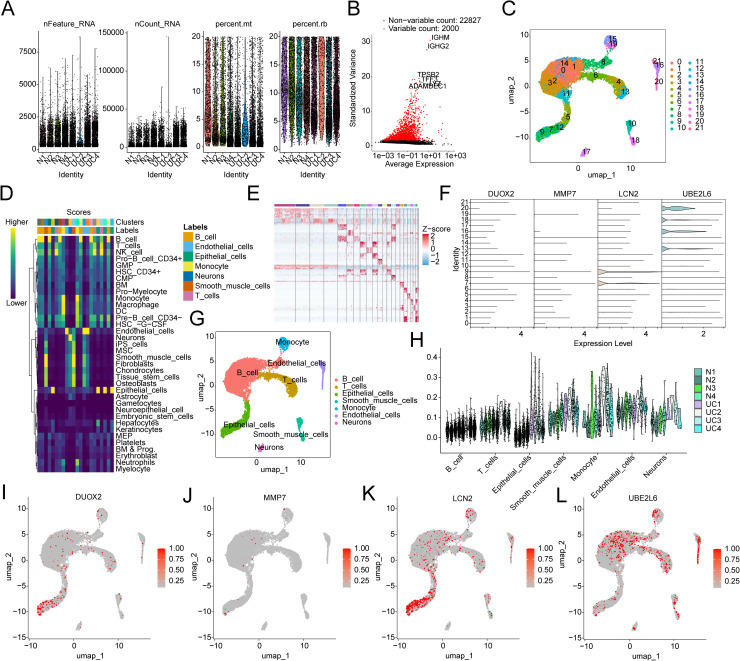
Single-cell sequencing analysis reveals the expression distribution of MMRG biomarkers in cell subtypes. **(A)** Quality control and preprocessing analysis of the samples. **(B)** Identification of the top 2000 highly variable genes. **(C)** Identification of cell subtypes. **(D)** Cell annotation based on SingleR. **(E)** Heatmap analysis of marker gene expression in cell subtypes. **(F)** Expression of MMRG biomarkers in 21 cell subtypes. **(G)** UMAP plot analysis of the 7 cell subtypes. **(H)** Expression analysis of MMRG signatures in the 7 cell subtypes. (**I-L)** Distribution and expression analysis of MMRG biomarkers in the 7 cell subtypes.

Subsequently, we analyzed the distribution and expression of MMRG diagnostic markers in different cell subtypes at the single-cell level. Based on the GSE231993 dataset, we extracted single-cell sequencing data from 4 UC samples and matched normal control samples. After quality control and preprocessing of all samples, we obtained 2000 highly variable genes for subsequent analysis (Fig 8A, B). Dimensionality reduction analysis of the quality-controlled data resulted in 21 cell clusters (Fig 8C). Using the SingleR algorithm for cell annotation, we evaluated the scores of each cell type and accurately identified 7 cell subtypes: B cells, T cells, epithelial cells, smooth muscle cells, monocytes, endothelial cells, and neurons (Fig 8D). A heatmap showing the marker genes for each cell cluster (Fig 8E). We then analyzed the expression distribution of 4 MMRG-related markers across the 21 cell clusters, which revealed significant expression of MMRG-related biomarkers in all 21 cell clusters (Fig 8F). The UMAP plot showed the distribution of these 7 cell subtypes (Fig 8G). Notably, in these 7 cell subtypes, significant expression of MMRG signatures was observed in each sample (Fig 8H). Further evaluation of the distribution of DUOX2, UBE2L6, LCN2, and MMP7 in the 7 cell subtypes using UMAP analysis showed that UBE2L6 was highly expressed in B cells, T cells, and endothelial cells, DUOX2 was highly expressed in epithelial cells, and LCN2 was most significantly expressed in epithelial cells (Fig 8I-L).

### qRT-PCR and western blot validation of MMRG biomarkers *in vitro*

**Fig 9:**
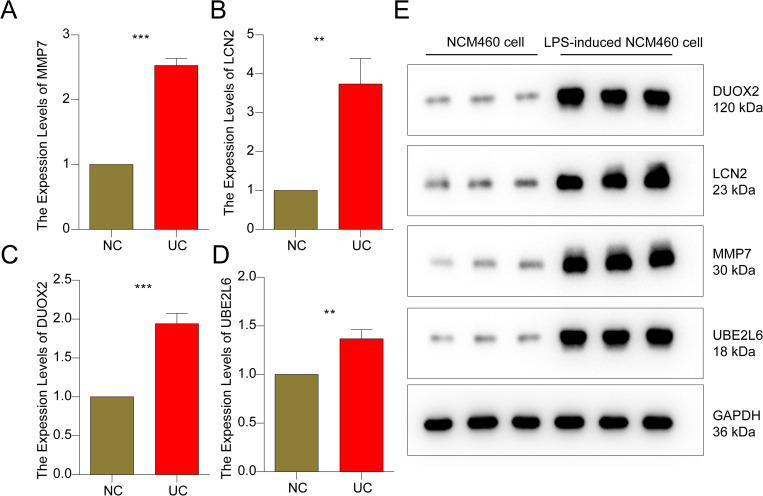
Expression detections of MMRG biomarkers in NCM460 cell lines and LPS-induced NCM460 cell lines. (**A-D)** The mRNA expression levels of 4 prognostic related MMRG biomarkers were validated by qRT-PCR in NCM460 cell lines and LPS-induced NCM460 cell lines. **(E)** Protein expressions of DUOX2, MMP7, UBE2L6 and LCN2 in NCM460 cell lines and LPS-induced NCM460 cell lines. Data are expressed as follows: mean ±  SD, * *p* < 0.05, **p < 0.01, ***p < 0.001 (n = 3).

We further verified the expression levels of these four MMRG biomarkers *in vitro* at NCM460 cell line and LPS-induced NCM460 cell line levels. In the LPS-induced NCM460 cell line, the mRNA expression levels DUOX2, UBE2L6, LCN2 and MMP7 were significantly overexpressed in comparison to normal NCM460 cell line (Fig 9A-D). Western blot was conducted to detect the protein expressions of screened biomarkers and the results was in line with qRT-PCR results (Fig 9E). These results partially validated the reliability of our bioinformatics analysis.

## Discussion

In this study, we screened UC-associated MMRG biomarkers and analyzed the molecular subtyping and immune infiltration status of UC. Single-cell sequencing analysis also supported the effect of MMRG on different cell components in UC patients. Our results support that UC patients can be divided into two subgroups with different immune-infiltration status based on MMRG and screen for four MMRG biomarkers. UC is associated with a lack of energy in the colon [17]. A previous metabolic analysis showed NAD^+^ metabolic dysregulation and altered mitochondrial status in UC patients [18]. This idea was also supported by the highest differential expression of genes related to mitochondrial function in the distal colon, where UC is usually affected [19]. Further proteomic analysis of the intestinal mucosa of UC patients showed that the down-regulated proteins included a group of proteins involved in energy production [20]. This is consistent with our results showing a correlation between MMRG and UC. In terms of specific mechanisms, it has been reported that the decrease of colon ATP level in UC patients may be related to the deficiency of short-chain fatty acids [21]. In a state of low intestinal energy, colon cells tend to adopt autophagy as an alternative energy source. The autophagy defects in UC can then cause further mitochondrial dysfunction [22]. Together with our results, these evidences suggest the importance of MMRG in the pathogenesis and treatment of UC.

Our results support the role of four MMRG biomarkers in UC. In 2015, a gene defect in DUOX2 was reported in patients with early-onset inflammatory bowel disease [23]. Further evidence suggests that overexpression of DUOX2 is involved in the regulation of toll-like receptor 4 (TLR4) in IBD [24]. As an innate immune receptor, TLR4 recognizes gram-negative LPS and triggers antimicrobial and pro-inflammatory responses [25,26]. Overexpression of TLR4 in colon epithelial cells of UC patients and its effect on UC have been confirmed [27,28]. In addition, DUOX2 was reported to be involved in forming an enzyme system that produces reactive oxygen species in active UC [29]. Therefore, DUOX2 may influence the development of UC through the above ways.

We provide evidence that LCN2 expression is elevated in UC. LCN2 is a low molecular weight protein released by activated neutrophils and intestinal epithelium [30]. LCN2 is associated with UC duration and cancer risk [31], which may be regulated by IL-17A, IL-22, and TNF-α [32]. It was reported that LCN2 is a key factor regulating ferroptosis in UC in vivo induction models [33]. Combined with the important role of ferroptosis in the regulation of intestinal epithelial homeostasis during the development of UC, the regulatory effect of LCN2 on UC is worthy of further study [34,35].

UBE2L6, a member of the proteasome system, was found to have a significant increase in protein mass in the mucosa of patients with IBD [36]. intestinal lamina propria macrophages (IMACs) maintained tolerance to food antigens and symbiotic bacterial flora [37]. However, increased UBE2L6 in IMACs in IBD patients can present antigenic peptides that support inflammation [36]. In addition, UBE2L6 can promote M1 macrophage polarization by activating STAT1 [38]. During the active phase of UC, macrophages in lamina propria of the intestinal wall are dominated by M1 [39]. M1 macrophages can destroy the epithelial barrier and induce apoptosis of epithelial cells, leading to excessive inflammation [40]. Conversely, macrophages with the M2 phenotype can help reduce intestinal inflammatory symptoms [41]. Our immune infiltration results also showed that UC patients had significantly higher macrophage levels. Therefore, the effect of UBE2L6 on intestinal macrophages deserves further study.

MMP is a group of enzymes that play an important role in the degradation and remodeling of extracellular matrix. In a mouse model of colitis with MMP-7 knockout, recruitment of neutrophils is significantly delayed [42]. There is a strong correlation between the expression of MMP-7 and the severity of UC in the colon immunohistochemical staining samples of UC patients [43]. Meanwhile, the expression of MMP-7 mainly in white blood cells also suggests its role in the development of inflammation [44]. Further studies have shown that MMP-7 damages the intestinal epithelial barrier by cutting Claudin-7, thereby increasing inflammation [45]. Although the above evidence shows the role of the above four biomarkers in UC, the current research is still insufficient, and the molecular pathway of the mechanism of action of biomarkers still needs to be further clarified.

Our results showed an increase in most immune cell components in UC patients compared to controls, and a higher level of immune cell differences between different UC subtypes. Immune processes are important in the development of UC. Goblet cells can form a barrier between the intestinal epithelium and the gut microbiome [46]. However, in UC patients, goblet cells decrease and lead to damage to the intestinal barrier and increased antigen exposure, triggering an inflammatory response [47]. Impaired balance between pro-inflammatory and anti-inflammatory processes allows chronic inflammation to develop [48]. In addition, changes in the balance of anti-inflammatory and pro-inflammatory cytokines during inflammation, which may lead to excessive immune response, may also be one of the reasons for the development of UC [49]. Our pathway enrichment results also suggest that a number of immunoinflammatory mediated pathways are significantly up-regulated in UC, further suggesting the role of innate and adaptive immune responses in the development of UC. It is of great clinical value to continue to explore the treatment of immune regulation in UC [50].

According to the above results, targeting mitochondria will provide new ideas for the treatment of current UC. Targeting specific MMRG to improve UC symptoms by regulating mitochondrial function has positive clinical value [51]. Currently reported potential mitochondria-related targets include PGC1a, PARK7 [52,53]. In addition, interventions in mitochondrial metabolic processes can induce changes in the gut microbiota and may affect resistance to certain therapy in UC patients [54]. However, due to individual heterogeneity, the pathogenesis of UC patients is different. Mitochondrial dysfunction was dominant in only some UC patients [55–57]. Therefore, further refinement of risk stratification methods is needed to identify UC patients who are sensitive to mitochondria-specific therapy.

We successfully analyzed the subtypes of UC patients based on MMRG, indicating possibility of individualized and precise therapy in UC treatment. Currently, based on factors including the composition of the gut microbiome, gene expression, Different types of IBD were proposed by non-coding RNA and other methods [58]. However, the clinical diagnosis, prognosis, and application of these classifications in all patients remains largely a subjective decision, with no operational objective classification method available [59]. Further research will not only help further understand the various molecular causes of IBD, but also help improve clinical trial design to develop more personalized disease management and treatment.

Due to the incomplete clinical data in public databases, the current data cannot be used to analyze the correlation between MMRG molecular subtypes and the clinical characteristics of UC patients. It is of clinical application value to further collect and sort out the clinical characteristics of UC samples to conduct correlation analysis with MMRG. In addition, we were unable to further explore the mechanism of action of the screened markers in UC. Although the current research on the targets we screened is insufficient, it is hoped that further *in vitro* and *in vivo* studies can verify the role of this screening target and clarify its mechanism.

## Supporting information

S1 Table The gene signature of MMRG. (XLSX)

S2 Table The gene primer sequences of MMRG biomarkers. (XLSX)

S1 File (ZIP)
