# Supplementary figures and images for: Integrated analysis and single-cell sequencing of mitochondrial metabolism related gene molecular subtype and diagnostic model in ulcerative colitis
Source: PLoS One. 2025 Mar 28;20(3):e0320010. doi: 10.1371/journal.pone.0320010 (PMC11952253; doi:10.1371/journal.pone.0320010)

DUOX2

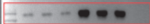

Supplement: S1 File — (ZIP) [file pone.0320010.s003.zip › WB/DUOX2.pdf]

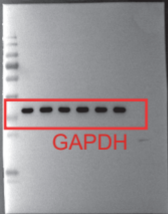

Supplement: S1 File — (ZIP) [file pone.0320010.s003.zip › WB/GAPDH.pdf]

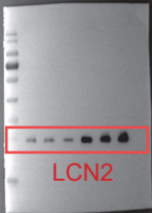

Supplement: S1 File — (ZIP) [file pone.0320010.s003.zip › WB/LCN2.pdf]

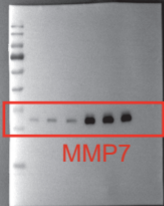

Supplement: S1 File — (ZIP) [file pone.0320010.s003.zip › WB/MMP7.pdf]

UBE2L6

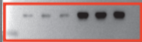

Supplement: S1 File — (ZIP) [file pone.0320010.s003.zip › WB/UBE2L6.pdf]
